# Effect of Quicklime Substitution for Cement on the Physical and Mechanical Properties of Autoclaved Fly Ash Aggregates via Hydrothermal Synthesis

**DOI:** 10.3390/ma18030707

**Published:** 2025-02-06

**Authors:** Dongyun Wang, Xuan Shen, Zhiyan Wang, Xiucheng Zhang, Xue-Fei Chen

**Affiliations:** 1 School of Architectural Engineering, Huanggang Normal University, Huanggang 438000, China; 2 Key Laboratory of Intelligent Health Perception and Ecological Restoration of Rivers and Lakes, Ministry of Education, Hubei University of Technology, Wuhan 430068, China; 3 School of Civil Engineering, Putian University, Putian 351100, China; 4 Huanggang Ecological and Renewable Resources Research, Huanggang 438000, China; 5 Hubei Province Prefabricated Green Building Technology Innovation Center, Huanggang 438000, China

**Keywords:** fly ash, aggregate, hydrothermal synthesis, physical and mechanical properties, cement, quicklime

## Abstract

Herein, we synthesized fly ash aggregates (FAAs) through a hydrothermal synthesis process utilizing fly ash, quicklime, and cement under saturated steam conditions at 180 °C. We systematically investigated the influence and mechanisms governing the physical and mechanical properties of autoclaved FAAs by incrementally replacing cement with quicklime in 5% equal mass intervals. Our results revealed that the substitution of cement with quicklime yielded lightweight aggregates (LWAs) exhibiting water absorption ranging from 1.33% to 22.88% after 1 h and 1.67% to 26.22% after 24 h, loose bulk densities between 847 kg/m^3^ and 1043 kg/m^3^, apparent densities spanning from 1484 kg/m^3^ to 1880 kg/m^3^, and cylinder compressive strengths varying from 11.9 MPa to 18.5 MPa. Notably, as the proportion of quicklime substitution for cement increased, there was a corresponding augmentation in water consumption during granulation, resulting in an elevated water–cement ratio ranging from 27.5% to 51.39%. This led to an enhancement in the water absorption of the FAAs, accompanied by a decrement in cylinder compressive strength and overall density. The hydration products, including tobermorite and calcium silicate hydrate, contributed to the creation of a dense microstructure within the FAAs. However, with higher quantities of quicklime replacing cement, the content of hydration products increased while the proportion of unreacted fly ash particles decreased significantly. The resultant weakening micro-aggregate effect emerged as a pivotal factor contributing to the observed decrement in the strength of these FAAs. The findings of this research are anticipated to provide significant theoretical insights and technical support for the selection of calcareous materials in the resource-recycling process of fly ash.

## 1. Introduction

Lightweight aggregates (LWAs) are defined as a type of particle characterized by a loose bulk density of less than 1200 kg/m^3^ or an apparent density below 2000 kg/m^3^ [1,2]. Owing to its lightweight, high-strength, and porous nature [3], concrete produced by substituting crushed stones with LWAs exhibits advantages such as reduced weight and enhanced strength [4,5,6,7,8,9]. Additionally, it provides thermal insulation, heat retention, and soundproofing properties, which collectively enhance transportation and construction efficiency [10,11]. Furthermore, the use of LWAs contributes to lower carbon emissions during both building construction and operation, thereby demonstrating significant market competitiveness in applications such as prefabricated buildings, high-rise structures, and large-span bridges. As early as the beginning of the 20th century, extensive research on lightweight aggregate concrete had been conducted both domestically and internationally, and it had been applied in engineering. For instance, in 1913, the United States used shale-expanded clay to produce lightweight aggregate concrete and built the Houston Shell Plaza Building. In 1968, the Nanjing Yangtze River Bridge, independently designed and constructed by China, adopted lightweight aggregate concrete made from fly ash for the entire deck of the highway bridge. This was the first time that lightweight aggregate concrete was applied to a super-large bridge in China, and the bridge is still in use today. In 2013, the “China Zun” project in Beijing partially utilized lightweight aggregate concrete. The project has 108 floors above ground and a total height of 528 m. It was successfully selected as one of the Top 100 Buildings of the New Era in China 2022 [12].

Based on the source of raw materials, LWAs can be categorized into artificial and natural products. Given that natural LWAs are a non-renewable resource with limited availability, the development of artificial LWAs with enhanced performance has become increasingly favorable. Artificial LWAs can further be classified into sintered and non-sintered types based on their preparation processes. Conventional sintered LWAs primarily utilize clay as a restrictive raw material and undergo sintering at temperatures exceeding 1000–1200 °C [13,14,15,16,17]. This process entails significant energy consumption, which poses challenges for environmental protection, energy conservation, and emission reduction. In contrast, non-sintered LWAs benefit from efficient solid waste treatment and eliminate the need for high-temperature sintering [18,19,20]. As a result, it demonstrates superior product performance while achieving objectives such as reducing carbon emissions, conserving natural resources, protecting ecological environments, and enhancing economic benefits. Autoclaved LWAs represent one category of non-sintered material. It is an innovative product derived from mixing, granulation, and autoclave curing using calcareous and siliceous raw materials [4,21,22,23].

Fly ash is an industrial by-product generated from coal-fired power plants. It consists of a silica–alumina glass-based material that incorporates volcanic ash. Under standard temperature curing conditions, the silica–alumina glass matrix reacts to form calcium silicate hydrate or calcium aluminate hydrate gel in an alkaline solution environment, imparting a certain degree of strength to the product. Consequently, many scholars frequently utilize fly ash as a raw material, incorporating specific amounts of cement or quicklime to produce burn-free pulverized coal ash LWAs at room temperature through a granulation process [24,25,26]. Most reports have concentrated on the room temperature curing process; however, investigations into the autoclave curing process for fly ash-based LWAs remain relatively scarce.

Cement and quicklime are two prevalent calcium raw materials utilized in autoclaved products [27,28]. The primary phases of ordinary Portland cement include tricalcium silicate and dicalcium silicate, which, upon contact with water at room temperature, generate a high CaO/SiO_2_ ratio C-S-H gel and Ca(OH)_2_. Under autoclaved curing conditions, the high CaO/SiO_2_ ratio C-S-H continues to engage in hydrothermal synthesis reactions with dissolved SiO_2_ present in the solution, leading to the formation of tobermorite and other hydro-calcium silicates [29]. The main phase of quicklime is CaO, which undergoes volumetric expansion when it comes into contact with water while releasing a significant amount of heat. Following digestion, Ca(OH)_2_ can be obtained. Under autoclaved curing conditions, Ca(OH)_2_ reacts with SiO_2_ and H_2_O to produce hydro-calcium silicates. It is evident that the hydrothermal synthesis processes for both cement and quicklime under autoclaved curing conditions are fundamentally similar. Both provide Ca^2+^ ions that participate in the calcium–silicon reaction, resulting in the formation of hydro-calcium silicates. However, under natural curing conditions, the hydration process of cement occurs more rapidly than that of quicklime. This disparity leads to differing effects on material performance between cement and quicklime, ultimately resulting in variations in physical and chemical properties within the autoclaved samples.

In view of the above, the present study utilizes fly ash as the silica raw material and selects quicklime as a substitute for cement. The hydrothermal synthesis and autoclaving processes are employed to produce fly ash aggregates (FAAs). This research determines the loose bulk density, apparent density, cylinder compressive strength, water absorption, mineral composition, microstructure, and loss on ignition of the FAAs in order to investigate the influence and mechanism of replacing cement with quicklime on the physical and mechanical properties of autoclaved FAAs. Ultimately, this study aims to provide a theoretical foundation and technical support for selecting calcium raw materials in the production of autoclaved FAAs.

## 2. Experimental Details

### 2.1. Materials

In synthesizing fly ash aggregates (FAAs), a selection of pivotal raw materials is employed, encompassing cement, quicklime, and fly ash. Precisely, the cement utilized in this synthesis is ordinary Portland cement (OPC) of grade 52.5, sourced from the reputable Jiangnan Onoda Cement Co., Ltd., situated in Nanjing, China. This specific grade of cement is chosen due to its optimal mechanical properties and chemical stability, which are crucial for the desired synthesis outcomes. The fly ash utilized in this process is procured from a thermal power plant located in Nanjing, China. Fly ash, being a by-product of coal combustion, possesses valuable pozzolanic properties that enhance the overall reactivity and strength of the synthesized material. The detailed chemical compositions of the fly ash, cement, and quicklime are systematically outlined in Table 1, providing a comprehensive understanding of their elemental makeup. Moreover, the quicklime used in this synthesis was acquired from Nanjing Changle Desiccant Co., Ltd., also located in Nanjing, China. Quicklime, primarily composed of calcium oxide (CaO), plays a crucial role in the neutralization and alkalinity adjustment of the reaction mixture. Notably, the effective CaO content of the quicklime employed in this study has been determined to be 90%, which underscores its high purity and reactivity, further contributing to the efficiency and effectiveness of the synthesis process.

### 2.2. Sample Preparation

**Figure 1:**
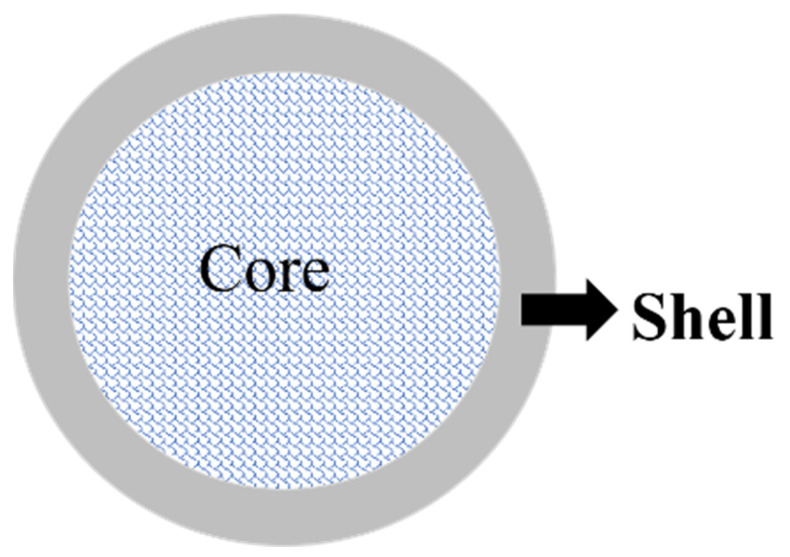
A schematic diagram of FAAs.

The fly ash aggregates (FAAs) encompass two fundamental constituents: the shell and the core, as depicted in Figure 1. Prior scholarly endeavors [22] have conclusively demonstrated that the thickness of the shell comprises approximately 4 wt.% of the core’s mass. The constituent materials utilized within the shell involve fly ash (FA) and cement, with a precise mass ratio of 85:15. In this investigation, it was imperative to ensure uniformity across all FAAs regarding both shell thickness and shell composition.

Preceding laboratory studies have revealed that when the cement content within the core is set at 35 wt.% and FA at 65 wt.%, the compressive strength of autoclaved FAAs attains its peak value. Consequently, a reference cohort designated as Q0 was established for this experiment, adhering to these proportions—specifically, 35 wt.% cement and 65 wt.% FA. Building upon this foundational understanding, while maintaining a constant dosage of FA at 65 wt.%, quicklime was introduced as a cement substitute, incremented by 5 wt.% intervals. This methodological approach is aimed at elucidating the effects of substituting cement with hydrated lime on the performance characteristics of autoclaved FAAs.

To facilitate a comprehensive understanding, the specific mix proportion designs for these materials have been meticulously detailed in Table 2. This tabulation serves as a reference and ensures reproducibility and transparency in the experimental procedure. By systematically altering the cement-to-quicklime ratio while keeping other variables constant, this study provides a robust analytical framework for assessing the impact of hydrated lime substitution on the mechanical properties of FAAs.

**Figure 2:**
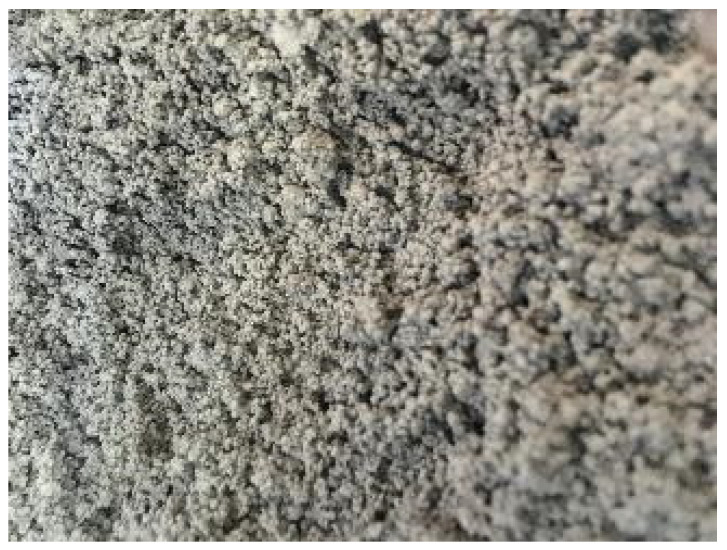
The photograph of the state of powdered materials mixed with water.

Regarding Table 2, the quicklime and fly ash (FA) were precisely weighed and uniformly blended, followed by adding water for digestion. The resultant mixture was sealed with plastic film and allowed to stand at ambient temperature for a minimum duration of 4 h to facilitate digestion. The quantity of water employed for digestion was recorded, and the digestion water ratio was subsequently calculated. Subsequently, cement was incorporated into the digested mixture within a mixer, and water was incrementally added until the powder components tended to agglomerate into fine particles, as depicted in Figure 2. The addition of water ceased once a wet powder mixture was achieved. At this juncture, the mass of water consumed during mixing was documented, and the mixing water ratio was computed. Thereafter, the wet powder mixture was transferred into a pelletizing pan and subjected to rotation. Upon reaching particle sizes ranging from 5 mm to 16 mm, dry powder from the shell layer was proportionally sprinkled onto the mixture, and rolling was continued for an additional 1–2 min to form raw fly ash aggregates (FAAs). After allowing these raw FAAs to rest under indoor conditions for 24 h, they were placed in a laboratory autoclave for curing.

The hydrothermal synthesis and autoclave curing protocol implemented in this study comprised the following steps: an increase in temperature from ambient to a curing temperature of 187 °C over a period of 3 h, followed by the maintenance of both temperature and pressure at 1 MPa for 10 h. Subsequently, the temperature was gradually reduced over a 2 h period back to ambient levels. Upon completion of the curing process, the FAAs were removed from the autoclave and placed in an oven until they reached a constant weight. Subsequently, their physical and mechanical properties were evaluated.

### 2.3. Testing

Drawing upon the standardized methodologies outlined in China [30] for lightweight aggregates and associated testing procedures, the characterization of fly ash aggregates (FAAs) was conducted based on the parameters of loose bulk density, apparent density, water absorption, and cylinder compressive strength. The pertinent calculations for these metrics are detailed in Equations (1)–(4).

(1) $$\rho_{1}=\frac{10^{3}\times m}{V_{1}}\;$$

The loose bulk density of the aggregates was ascertained using Equation (1), where ρ_1_ signifies the loose bulk density (kg/m^3^), m represents the dry mass of the aggregates (g), and V_1_ denotes the volume of the container (cm^3^).

(2) $$\rho_{2}=\frac{10^{3}\times m}{V-500}$$

The apparent density of the aggregates was determined via Equation (2), where ρ_2_ indicates the apparent density (kg/m^3^), m signifies the dry mass of the aggregates (g), V represents the volume as indicated by the final reading of the graduated cylinder (cm^3^), and 500 is the volume of the additional water utilized in the graduated cylinder (cm^3^).

Water absorption was defined as the differential mass between dry aggregates and those saturated with water after immersion for durations of 1 h and 24 h. The formulations for 1 h and 24 h water absorption are outlined in Equation (3), where W_i_ denotes the water absorption of the aggregates (%), with i = 1 representing the 1 h water absorption (%) and i = 24 signifying the 24 h water absorption (%), m is the dry mass of the aggregates (g), and m_i_ indicates the mass of the aggregates subsequent to immersion (g), where m_1_ pertains to the mass after 1 h immersion (g) and m_24_ pertains to the mass after 24 h immersion (g).

(3) $$W_{i}=\frac{100\%\times \left(m_{i}-m\right)}{m}$$

FAAs with diameters spanning from 5 to 16 mm were placed within a cylinder possessing an inner diameter of 56.9 mm and a height of 120 mm, upon which a uniform load was applied. The pressure value was documented when the punch indentation depth reached 20 mm. This measurement reflects the cylinder compressive strength of the FAAs, calculated in accordance with Equation (4).

(4) $$f=\frac{P}{A}$$

Conforming to the specifications outlined in standard JC/T 478.2-2013 [31], the loss on ignition (LOI) of the FAAs was determined with the calculation formula presented in Equation (5). Here, LOI signifies the percentage of weight loss experienced by the material subsequent to drying at 105 °C and maintaining at 950 °C for 20 min; m_0_ represents the mass of the crucible (g); m_1_ denotes the mass of the sample prior to ignition (g); and m_2_ signifies the combined mass of the sample and crucible post-ignition (g).

(5) $$\;\mathrm{LOI}=\frac{m_{2}-m_{0}}{m_{1}}\times 100\%\;$$

The chemical composition of the raw materials was characterized utilizing a PW1710 X-ray fluorescence spectrometer manufactured by Philips (Amsterdam, The Netherlands). The particle size distribution was evaluated with a BT-9300S laser particle size analyzer, while the specific surface area was quantified using a V-Sorb 28009 model (Hefei, China). The crystalline phases of the samples were examined via X-ray diffraction (XRD) with CuKα radiation (λ = 1.542 Å) at an accelerating voltage of 40 kV and a current of 40 mA, employing a Bruker D8 Advance apparatus (Karlsruhe, Germany). Microstructural morphologies of the samples were captured using scanning electron microscopy (SEM) with a Model Quanta 250FEG manufactured by FEI (Veldhoven, The Netherlands). The accelerating voltage during the SEM test is 20 kV. The surface of FAAs was sputtered with gold layers in order to improve the quality of the images.

## 3. Results and Discussion

### 3.1. Particle Size Distribution of Raw Materials

The granularity and specific surface area of raw materials exhibit a complex interplay with several critical factors in the manufacturing process, including the reaction kinetics [32], the water demands during the granulation phase [33], the physical attributes of the resultant aggregates [34], and the efficacy of molding operations [35]. Specifically, finer raw materials correspond to a larger specific surface area, which translates into an increased number of contact interfaces among particles, more thorough reactions, and a greater abundance of hydration products. These phenomena collectively contribute to accelerated strength development in autoclaved products [32]. Furthermore, the utilization of finer raw materials enhances molding performance and facilitates the production of aggregates characterized by larger particle sizes [35].

**Figure 3:**
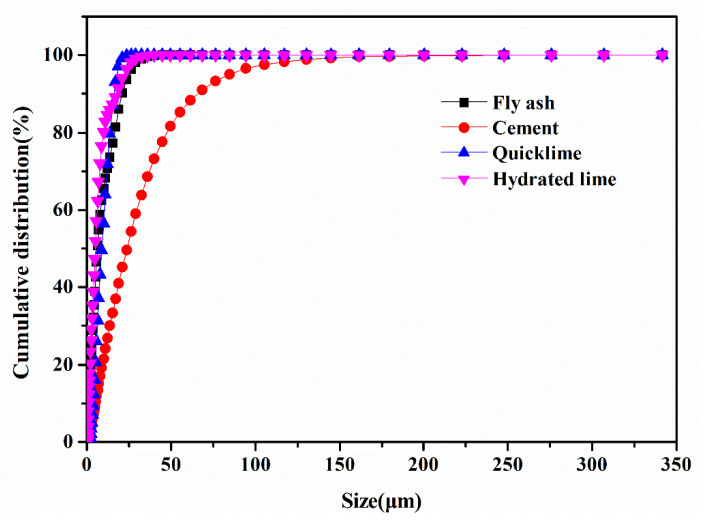
The particle sizes of quicklime, hydrated lime, cement, and fly ash.

To gain a deeper understanding of these relationships, a quantitative assessment of the particle sizes of fly ash, quicklime, hydrated lime (resulting from the post-water digestion of quicklime), and cement was conducted. The outcomes of this analysis are graphically represented in Figure 3. It elucidates that the particle size distribution of fly ash is quite broad, with notable concentration peaks observed within the ranges of 6–8 μm and 18–20 μm. The median diameter (D50), measured at 6.38 μm, suggests that fly ash particles are relatively small in size, which is advantageous for the pelletization process. On the other hand, quicklime exhibits the highest concentration within the range of 12–14 μm, with a median diameter (D50) of 9.01 μm. This indicates that its fine particle size enhances its capacity to produce well-dispersed hydrated lime upon hydration.

Hydrated lime displays a broad particle size distribution, characterized by significant concentration areas spanning four specific intervals: 1–3 μm, 2–4 μm, 6–8 μm, and 20–22 μm. The median diameter (D50) for hydrated lime is approximately 5.01 μm, indicating a superior pelletization capability following the conversion of quicklime to hydrated lime. In contrast, cement has a median diameter (D50) estimated at around 23.67 μm, which is approximately 18.66 μm larger than that of hydrated lime. During the granulation process, quicklime undergoes hydration to form hydrated lime. Consequently, the fly ash-based aggregates (FAAs) are composed of two or three materials among hydrated lime, cement, and fly ash. Given that hydrated lime possesses a larger specific surface area and smaller particle size compared to cement, an increase in the substitution ratio of quicklime for cement—from 0% to 5%, 10%, 15%, 20%, 25%, and 30%, respectively—results in a corresponding rise in the water consumption rate for the granulation of the FAAs. Specifically, the water consumption rate increases from 27.5% to 31.45%, 32.18%, 36.17%, 41.36%, 47.91%, and 51.39%, respectively. This variation in water consumption during the granulation process has a profound impact on the physical and mechanical properties of the resultant FAAs.

In summary, the granularity and specific surface area of raw materials are pivotal factors influencing the granulation process, the physical attributes of aggregates, and the overall performance of the final products. By controlling these parameters, the production process can be optimized, and desired product characteristics can be achieved.

### 3.2. Physical and Mechanical Properties of FAA

#### 3.2.1. Water Absorption

**Figure 4:**
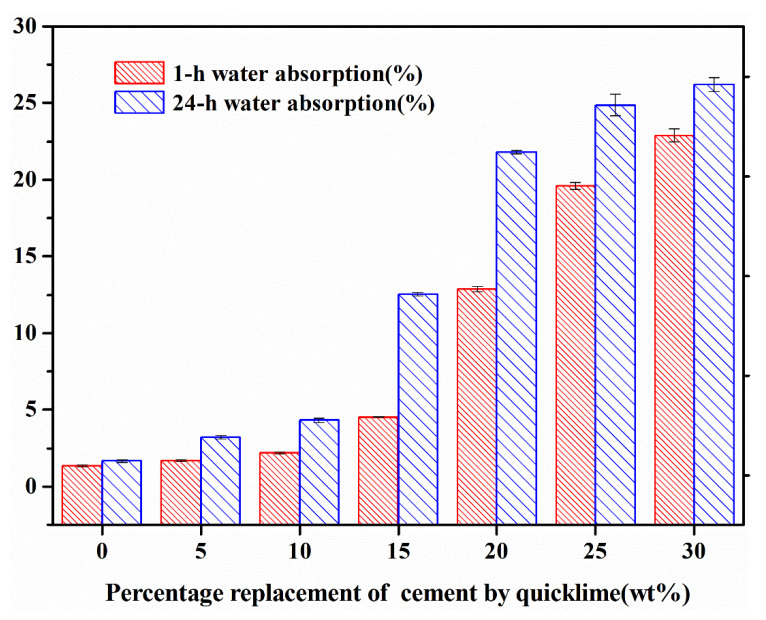
The water absorption of fly ash aggregates (FAAs).

Figure 4 delineates the water absorption characteristics of fly ash aggregates (FAAs) as a function of the substitution level of quicklime for cement. A discernible trend emerges, where both the one-hour and 24 h water absorption rates of the FAAs exhibit a consistent upward trajectory with an increasing substitution of quicklime for cement. This observation implies that a heightened substitution of quicklime leads to a decrement in the density of the FAAs while concurrently augmenting their internal porosity.

During the autoclaving phase, the raw materials comprising Ca(OH)_2_, SiO_2_, and Al_2_O_3_ undergo hydrothermal synthesis reactions with water, resulting in the formation of calcium silicate hydrate (C-S-H), which acts as a filler within existing cavities or voids. This filling effect is beneficial in terms of structural integrity. Additionally, as the proportion of quicklime substituted for cement escalates, there is a proportional augmentation in the hydration product content, as corroborated by X-ray diffraction (XRD) and scanning electron microscope (SEM) analyses detailed in Section 3.3 and Section 3.4. In theoretical terms, such an increase in hydration product content would be expected to enhance the density of the material and consequently decrease its water absorption.

However, the findings of this study reveal a counterintuitive trend: within the context of FAAs, water absorption progressively increases with higher levels of quicklime substitution for cement. This anomaly can be attributed to the elevated water consumption during the granulation process, which intensifies with increased quicklime replacement, as evidenced in Table 2. During static curing processes, the evaporation of water leads to the formation of cavities within the green pellets, exacerbating initial structural defects and ultimately diminishing the overall density of the FAAs.

Consequently, it becomes evident that the detrimental impact stemming from granulation-related water consumption on the water absorption of the FAAs overshadows any beneficial filling effects attributable to the hydration products formed via hydrothermal synthesis reactions. Therefore, it is concluded that as the substitution of quicklime for cement increases, both the one-hour and 24 h water absorption rates of the FAAs exhibit a gradual and progressive elevation. This underscores the need for further research to optimize the balance between quicklime substitution and water absorption in the production of FAAs.

In Figure 4, the examination revealed that when the substitution level of quicklime for cement ranged from 0 to 15%, the one-hour water absorption values of fly ash aggregates (FAAs) Q0 to Q15 were relatively modest, spanning from 1.33% to 4.53%, which remained beneath the critical threshold of 5%. Zhu et al. [36] corroborated this observation by affirming that at lower saturation levels, lightweight aggregates (LWAs) continue to absorb moisture, thereby diminishing both slump and time-dependent loss in LWA concrete. Consequently, this affects the pumpability and workability of the concrete mixture during placement. Therefore, LWAs typically necessitate pre-wetting for a duration of one hour prior to application [37]. Conversely, Li et al. [38] demonstrated that for LWAs exhibiting low water absorption, specifically those with a one-hour absorption rate below 5%, pre-wetting for merely half an hour can significantly enhance the segregation resistance and pumpability of fresh LWA concrete mixtures.

When the substitution amount of quicklime for cement increased within the range of 20% to 30%, the FAAs Q20 to Q35 exhibited markedly higher water absorption values. Specifically, their one-hour absorption values ranged from 12.88% to 22.88%, and their 24 h absorption values ranged from 21.8% to 26.22%. High-water-absorption LWAs impart a notable internal curing effect on cement matrices characterized by low water-to-cement ratios. This phenomenon enhances the interfacial transition zone strength and substantially improves the overall mechanical properties of concrete, thereby alleviating issues related to drying shrinkage and self-shrinkage. Consequently, high-water-absorption LWAs hold promise as internal curing agents in high-performance concrete formulations with reduced water–binder ratios [39,40].

These findings suggest that through precise manipulation of both cement and quicklime dosages, it is feasible to regulate the water absorption characteristics of FAAs. Increasing cement content may lead to reduced water absorption, whereas augmenting quicklime content tends to elevate absorption levels. This dual-pronged approach enables the effective fulfillment of specific performance criteria. Cioffi et al. [41] similarly reported comparable findings in traditional non-sintered FAAs, revealing that LWAs produced using quicklime combined with fly ash displayed higher water absorption than those manufactured using conventional cement–fly ash combinations. This underscores the significance of balancing cement and quicklime dosages to achieve optimal water absorption characteristics in FAAs.

#### 3.2.2. Cylinder Compressive Strength

**Figure 5:**
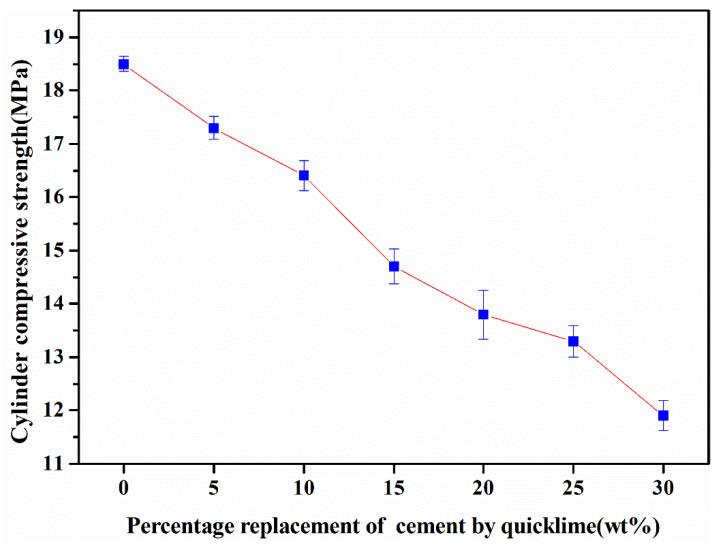
The cylinder compressive strength of fly ash aggregates (FAAs).

Figure 5 illustrates the variation in cylinder compressive strength exhibited by fly ash aggregates (FAAs). It is evident from the graphical representation that the compressive strength of these aggregates decreases progressively from 18.5 MPa to 11.9 MPa. This decrement can be logically attributed to the elevated CaO content in quicklime, which constitutes approximately 90%, markedly surpassing the 64% present in conventional cement. As the proportion of quicklime substitution for cement increases, an excessive accumulation of Ca^2+^ ions occurs in the solution under autoclaving conditions. Consequently, an abundance of Ca(OH)_2_ reacts with SiO_2_, leading to the formation of hydration products characterized by high alkalinity and low mechanical strength [42]. This chemical reaction sequence ultimately results in a decrement in the compressive strength of the FAAs.

Furthermore, an analysis of Table 2 reveals an intriguing trend: as the magnitude of quicklime substitution for cement rises from 0% to 30%, the water-to-binder (comprising cement, quicklime, and fly ash) ratio escalates from 0.275 to 0.5139. This augmentation in the water-to-binder ratio contributes to an increase in the superfluous water content within the FAA matrix. The excess water, upon vaporization during the green body stage, creates pores, which diminish the effective cross-sectional area of the FAAs tasked with bearing loads. Consequently, stress concentrations develop in the vicinity of these pores when subjected to external loads, leading to a further decrement in the compressive strength of the FAAs as the water-to-binder ratio increases. Notably, the attenuation of sample strength due to an elevated water-to-binder ratio is a phenomenon commonly observed in ordinary concrete as well. Also, they could partially explain how the cement replacement by quicklime can lead to inner microstructural changes [43], causing a decrease in the mechanical properties at the nanoscale level [44].

Importantly, Figure 5 also underscores that the cylinder compressive strength of the prepared FAAs, ranging from 11.9 MPa to 18.5 MPa, is notably high. According to the specifications outlined in GB/T 17431.1-2010 [1], aggregates possessing a cylinder compressive strength exceeding 6.5 MPa are classified as high-strength lightweight aggregates (LWAs). Consequently, all the FAAs fabricated in this study qualify as high-strength LWAs, highlighting their potential for utilization in applications requiring materials with superior mechanical properties.

#### 3.2.3. Loose Bulk Density and Apparent Density

**Figure 6:**
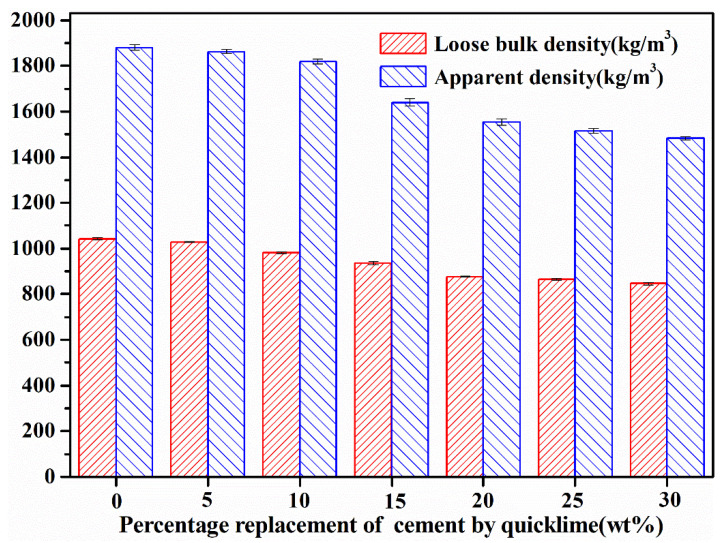
The loose bulk density and apparent density of fly ash aggregates (FAAs).

Figure 6 presents a comprehensive analysis of the loose bulk density and apparent density of fly ash aggregates (FAAs). As the substitution rate of lime for cement escalates, a discernible decrement is observed in both the loose bulk density and apparent density of the FAAs. This phenomenon can be rationalized by the fact that quicklime undergoes hydration to form hydrated lime during the preparation process of the FAAs. Consequently, the composition of 1 m^3^ of FAAs encompasses hydrated lime, cement, and fly ash. It is pertinent to note that hydrated lime possesses a density of 2240 kg/m^3^, which is inferior to the density of cement, which stands at 3100 kg/m^3^. Therefore, as the substitution rate of quicklime for cement increases, there is a gradual decrement in the overall density of the FAAs.

Moreover, it further elucidates that the water absorption capacity of the FAAs diminishes as the proportion of quicklime replaces cement augments. This trend suggests a reduction in the density of the FAAs and an augmentation in porosity. This phenomenon represents another pivotal factor contributing to the decrement in density observed in the FAAs. The loose bulk density and apparent density of the FAAs are quintessential characteristics of lightweight aggregates (LWAs), serving as critical indicators for the successful fabrication of lightweight aggregate concrete. According to the specifications outlined in GB/T 17431.1-2010 [1], a material can be classified as a light aggregate if its loose bulk density is less than 1200 kg/m^3^ or its apparent density is below 2000 kg/m^3^. In this study, the loose bulk density of the FAAs ranged from 847 to 1043 kg/m^3^, comfortably falling below the threshold of 1200 kg/m^3^. Additionally, the apparent density varied from 1484 to 1880 kg/m^3^, remaining well under the limit of 2000 kg/m^3^. Consequently, all the samples produced in this research can unequivocally be categorized as LWAs, highlighting their potential for utilization in applications requiring materials with reduced weight and density.

### 3.3. Crystalline Phases and Loss on Ignition Analysis

**Figure 7:**
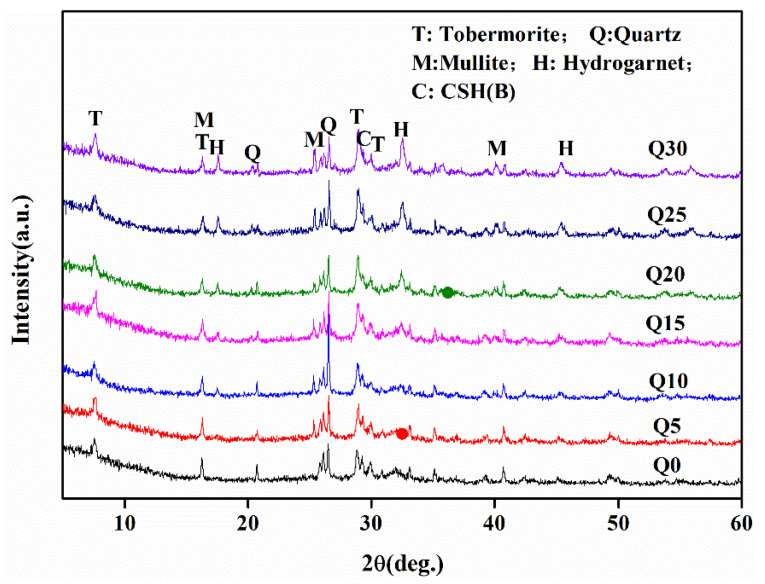
XRD patterns of fly ash aggregates (FAAs).

Figure 7 offers a detailed examination of the X-ray diffraction (XRD) patterns of fly ash aggregates (FAAs), providing valuable insights into their mineralogical composition. Upon scrutiny, it becomes evident that the mineral phases present within the FAAs encompass hydration products such as tobermorite (5CaO·6SiO_2_·5H_2_O, with characteristic interplanar spacings of 11.3 Å, 3.08 Å, and 2.98 Å), hydro-garnet (3CaO·Al_2_O_3_·SiO_2_·4H_2_O, exhibiting interplanar spacings of 5.05 Å and 2.75 Å), residual mullite (3Al_2_O_3_·2SiO_2_), and quartz (SiO_2_).

It reveals that samples ranging from Q0 to Q30 display a diffuse diffraction peak at 2θ = 29.2°, indicative of the presence of poorly crystallized hydration phases. Specifically, this peak suggests the existence of amorphous calcium silicate hydrate (CSH(B)), which possesses primary characteristic peaks at 3.05 Å and falls within the compositional range of 0.8~1.5 CaO·SiO_2_·nH_2_O. This observation underscores the fact that incorporating up to 30% by mass of quicklime into cement does not significantly alter the mineral phase composition of the FAAs. Furthermore, the presence of tobermorite within the FAAs is particularly noteworthy. As a mono-alkali hydration product, the tobermorite contributes to the high strength of the FAAs, as demonstrated in Figure 5. This attribute underscores the potential of these aggregates for use in applications requiring materials with robust mechanical properties.

Furthermore, as delineated in Table 1, the SiO_2_ content in fly ash is observed to be 53.47%, whereas the CaO content is 2.98%. In contrast, cement exhibits a SiO_2_ content of 20.3% and a CaO content of 64%. Notably, the effective CaO content in quicklime attains a level as high as 90%. When quicklime is utilized as a substitute for cement at rates of 20%, 25%, and 30%, respectively, the calcium–silicon ratios for the fly ash aggregates (FAAs) Q20, Q25, and Q30 can be approximated as 0.84, 0.90, and 0.96, based on the chemical composition of the constituent raw materials. These ratios approach or even surpass the threshold value of 0.83, theoretically indicating that all SiO_2_ should participate in reactions leading to the formation of tobermorite.

However, quartz diffraction peaks remain distinctly visible in the fly ash-based clay aggregates Q20 through Q30. This observation suggests that the reaction of quartz is incomplete. Consequently, an abundance of Ca^2+^ ions is present, with the excess Ca^2+^ ions contributing to the formation of high-alkali, low-strength hydration products, as outlined in previous research [42]. This phenomenon has an adverse impact on the mechanical strength of the aggregates, as evidenced in Figure 4. These findings underscore the complexity of the hydration reactions occurring within the FAAs and the need for careful consideration of raw material compositions to optimize the strength properties of the resulting aggregates.

Furthermore, an examination of Table 1 reveals that fly ash contains 53.47% SiO_2_ and 2.98% CaO, whereas cement comprises 20.3% SiO_2_ and 64% CaO. The effective CaO content in lime is notably high at 90%. When quicklime is substituted for cement at rates of 20%, 25%, and 30%, respectively, the calcium–silicon ratios of the fly ash aggregates (FAAs) Q20, Q25, and Q30 can be deduced to be approximately 0.84, 0.90, and 0.96, respectively, based on an analysis of the raw material’s chemical compositions. These ratios are found to be in close proximity to or even exceed the threshold value of 0.83, suggesting theoretically that all SiO_2_ should engage in reactions conducive to the formation of tobermorite.

However, as evident from Figure 6, the quartz diffraction peaks of the FAAs Q20 to Q30 persist, indicating that the quartz has not undergone a complete reaction. This observation implies an abundance of Ca^2+^ ions in the system. The presence of excess Ca^2+^ ions facilitates the formation of high-alkali, low-strength hydration products, as documented in previous studies [42,45]. Consequently, this has a detrimental impact on the mechanical strength of the aggregates. These findings underscore the need for a meticulous examination of the raw material compositions and their interactions during the hydration process to optimize the strength properties of the resultant fly ash aggregates.

The comprehensive analysis depicted in Figure 7 elucidates a significant trend: as the proportion of quicklime substituting for cement escalates, the diffraction peak associated with CSH(B) undergoes a progressive broadening (Figure 7). This phenomenon suggests that an elevated calcium source content promotes the formation of CSH(B), which in turn continues to engage in reactions with silicate ions present in the aqueous solution, ultimately fostering the generation of tobermorite. Additionally, there exists a direct correlation between an augmentation in the replacement ratio of quicklime for cement and an enhancement in the intensity of tobermorite diffraction peaks.

In conditions where an abundance of Ca^2+^ ions is prevalent, the presence of water garnet phases within the fly ash aggregates (FAAs) Q0 to Q30 is observed. This occurrence is attributed to the excessive concentration of Al_2_O_3_ in the fly ash. Furthermore, as the proportion of quicklime replacing cement increases, a corresponding intensification in the intensity of hydro-garnet diffraction peaks is discernible. These findings underscore the intricate interplay between the calcium source content, the silicate ions in solution, and the mineralogical composition of the fly ash, which collectively influence the formation and characteristics of the hydration products within the FAAs.

As illustrated in Figure 7, the mineralogical composition of the fly ash aggregates (FAAs) primarily comprises quartz, mullite, tobermorite, and hydro-garnet. Quartz and mullite exhibit remarkable stability in their physical and chemical properties, remaining unaffected by volatile products even at elevated temperatures. Conversely, tobermorite and hydro-garnet contain varying quantities of chemically bound water and hydroxyl groups, which undergo dehydration processes below 300 °C and dehydroxylation at approximately 750 °C [46], respectively [38]. Consequently, the FAAs synthesized in this study will undergo significant mass changes due to the dehydration and dehydroxylation processes during firing at 900 °C.

**Figure 8:**
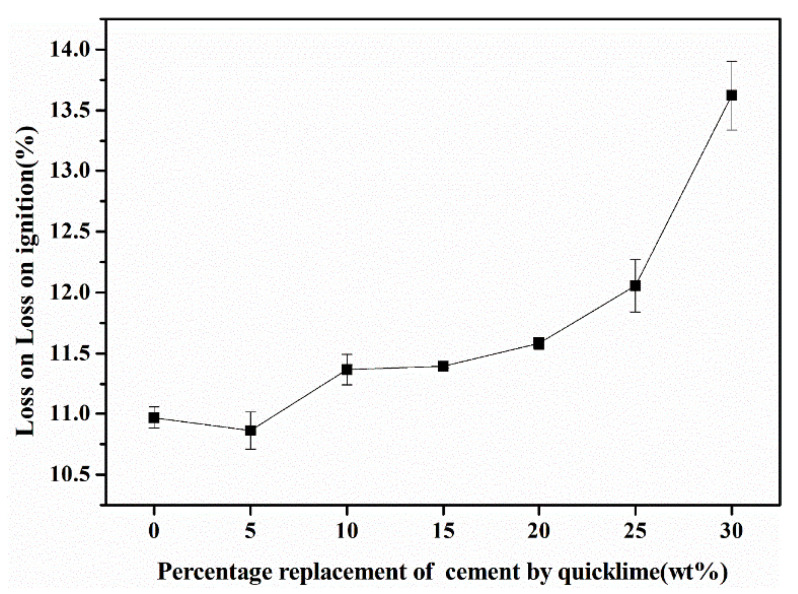
The loss on ignition of fly ash aggregates (FAAs).

To quantify the content of chemically bound water within the hydration products, mass loss measurements were conducted. These measurements provide valuable insights into the hydration product content, as presented in Figure 8. The results indicate that as the proportion of quicklime substituting for cement increases, there is a corresponding gradual augmentation in the mass loss content. This observation suggests that the quantity of hydration products within the slag-based clay aggregates also increases with higher levels of limestone substitution for cement. This finding is consistent with the X-ray diffraction (XRD) peak intensity results, which demonstrate enhanced intensities for both tobermorite and hydro-garnet as the quicklime replacement levels escalate.

Furthermore, an increase in hydration product content implies greater participation of fly ash in the hydration reactions, leading to a concurrent reduction in the quantity of unreacted fly ash particles. This decrease in unreacted fly ash particles diminishes the micro-aggregate effects, ultimately resulting in a gradual decrement in the strength of the FAAs (Figure 5). These observations underscore the intricate relationship between the mineralogical composition, hydration product content, and mechanical properties of the FAAs, emphasizing the importance of careful material selection and processing parameters to optimize their performance.

### 3.4. Microstructure Analysis

**Figure 9:**
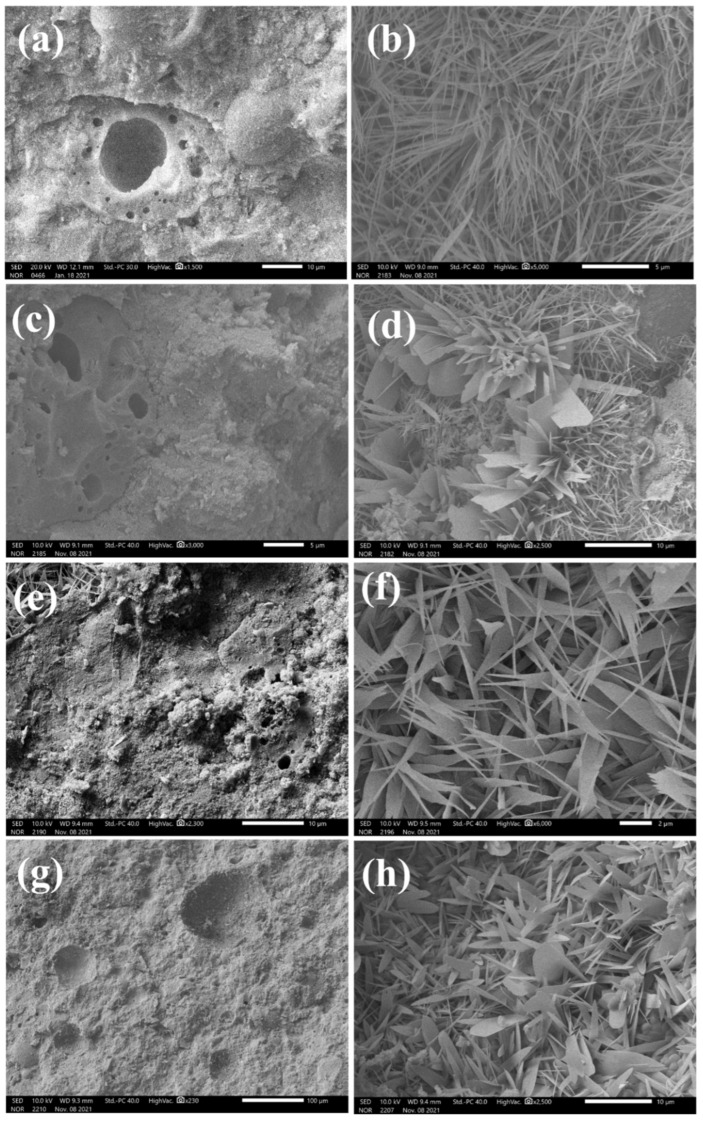
Micrographs of fly ash aggregates (FAAs): (**a**,**b**) Q0; (**c**,**d**) Q5; (**e**,**f**) Q15; (**g**,**h**) Q25.

Figure 9 presents scanning electron microscope (SEM) images of fly ash aggregates (FAAs) Q0, Q5, Q15, and Q25, offering a detailed microstructural examination of these materials. In Figure 9a,c,e,g, the SEM images reveal a dense microstructure for the FAAs, indicating that the calcium–silicon reaction under steam pressure generates sufficient hydration products to effectively occupy the pores and voids within the aggregates. This dense microstructure is a critical factor contributing to the high unconfined compressive strength demonstrated by these aggregates, as evidenced in Figure 4.

Additionally, the SEM images in Figure 9a,c,e, and g exhibit unreacted spherical fly ash particles, which serve as micro-aggregates within the FAAs. These particles are a primary source of strength for the aggregates, contributing to their overall mechanical properties. The presence of these spherical particles further highlights the complex interplay between the raw materials and the hydration reactions during the synthesis process.

In Figure 9b,d,f,h, needle-like hydration products are clearly visible and identified as tobermorite, consistent with the X-ray diffraction (XRD) analysis results presented in Figure 7. The interlocking nature of tobermorite plays a significant role in enhancing the overall strength of the fly ash clay aggregates. The needle-shaped morphology of tobermorite creates a robust, interconnected network that effectively transfers loads and enhances the mechanical resistance of the aggregates.

Notably, Figure 9e illustrates that needle-shaped tobermorite is present within the pores of the aggregates, providing a filling effect that mitigates initial defects and enhances the overall structural integrity of the FAAs. This filling effect contributes to the excellent mechanical properties of the aggregates, further underscoring the importance of the hydration reaction and the resulting microstructure in determining the performance of these materials. Overall, the SEM images presented in Figure 9 provide valuable insights into the microstructural characteristics of the FAAs and their relationship to their mechanical properties.

### 3.5. Overall Discussion

Through the particle size distribution, physical and mechanical properties, crystalline phases, microstructure, and loss on ignition analysis, we can find the following.

First, it is the effect of quicklime on FAA Properties. During the preparation of raw FAAs, quicklime undergoes slaking to produce hydrated lime, resulting in a reduction in the median diameter (D50) from 9.01 μm to 5.01 μm. This refinement of particle size significantly impacts the subsequent process steps. As the proportion of quicklime replacing cement increases, the demand for water in pellet formation escalates. This heightened water requirement leads to an augmentation in the water absorption capacity of the FAAs, a decrement in both loose bulk density and apparent density, and a notable decrease in cylinder compressive strength. The interplay between particle size refinement and water content is crucial in determining the final mechanical properties of the aggregates.

Second, it is the role of hydration products in microstructure density and strength. The filling effect imparted by hydration products, such as tobermorite and hydro-garnet, is indispensable in achieving a dense microstructure within the FAAs. However, as the substitution ratio of quicklime for cement increases, the content of these hydration products progressively rises. This trend leads to a decrease in the number of unreacted fly ash particles, thereby diminishing the micro-aggregate effect. This reduction in unreacted particles further contributes to the decline in cylinder compressive strength observed in the FAAs, highlighting the delicate balance between hydration product content and micro-aggregate effect on overall strength.

Third, it is the physical and mechanical properties of FAAs in compliance with standards. The water absorption of the FAAs exhibits a range from 1.33% to 22.88% after 1 h and 1.67% to 26.22% after 24 h. These values underscore the importance of considering water absorption in the design and application of FAAs. The apparent density of the FAAs spans from 1484 kg/m^3^ to 1880 kg/m^3^, while the loose bulk density ranges between 847 kg/m^3^ and 1043 kg/m^3^. These measurements demonstrate that the FAAs comply with the lightweight requirements specified in GB/T 17431.1-2010, affirming their suitability for lightweight construction applications. The cylinder compressive strength of the FAAs falls within the range of 11.9 MPa to 18.5 MPa, meeting the criteria for high-strength lightweight aggregates (LWAs) as outlined in GB/T 17431.1-2010. This finding underscores the potential of the developed FAAs in high-performance structural applications.

## 4. Conclusions

Through the application of a low-energy hydrothermal synthesis technique coupled with steam curing, we have successfully synthesized fly ash aggregates (FAAs) via calcium–silicon reactions. The dosage of quicklime replacing cement has a significant influence on the technological properties of the FAAs. Increasing proportions of quicklime has been shown to clearly increase water absorption and decrease bulk densities. On the other hand, quicklime addition entails a reduction in cylinder compressive strength due to increased porosity and the weakening of the micro-aggregate effect. The FAAs are well conformed to Chinese Standard (GB/T 17431.1-2010). The synthesis of FAAs through a low-energy hydrothermal technique combined with steam curing offers a promising approach for producing lightweight, high-strength aggregates with a dense microstructure.

In the future, we will apply FAAs as aggregate in high-performance concrete with a low water–cement ratio, explore the internal curing mechanism of FAAs and the mechanical properties and durability of FAAs concrete, and provide theoretical value and technical support for the application of FAAs.

## Figures and Tables

**Table 1:** Chemical composition of raw materials (wt.%).

Component	SiO_2_	Al_2_O_3_	Fe_2_O_3_	CaO	MgO	K_2_O	L.O.I.	Others
Cement	20.30	5.61	3.25	64.00	1.17	-	2.49	3.18
Fly ash	53.47	30.48	4.73	2.98	0.95	-	1.45	5.94

Note: LOI—loss of ignition.

**Table 2:** Mix proportions and water consumption of fly ash aggregates (FAAs) (wt.%).

Mix.	Core			Shell		Shell/Core	Water		
	C	QL	FA	FA	C		W1	W1	W3
Q0	35	0	65	85	15	4	0.00	27.50	27.50
Q5	30	5	65			4	5.70	25.75	31.45
Q10	25	10	65			4	7.00	25.18	32.18
Q15	20	15	65			4	16.99	19.18	36.17
Q20	15	20	65			4	17.43	23.93	41.36
Q25	10	25	65			4	18.74	29.17	47.91
Q30	5	30	65			4	20.39	31.00	51.39

Note: C—cement, QL—quicklime, FA—fly ash, Shell/Core—mass ratio between the shell and the core, W1—the ratio of water consumption utilized for quicklime digestion to the combined mass of the quicklime and fly ash, W2—the ratio of water consumption used for mixing to the combined mass of the quicklime and fly ash, W3—the sum of W1 and W2.

## Data Availability

The original contributions presented in this study are included in the article. Further inquiries can be directed to the corresponding authors.
